# 

**Published:** 1884-04

**Authors:** 


					CONTENTS:
Art. IV.-
-On the Relations between Dental Lesions and Diseases 01
By Henry Power, F. R. C. S.,
Atk. II -
-Pre-Natal Influences as Affecting the Teeth.
By Geo. H. Cut>
Art. III.-
-An Appeal for a Na-ional Deutal Association...
The Dental Bill of tbe State < f JSevv Jersey..,
EDITORIAL, ETC.
Dental Legislation?Opinions For and Against.
A New Steel.
The Soul hern Denial Association.
Illinois State Dental Asssociation..
OBITUBRY.
In Memoriam.
?Henry Hokirt Keech,
M. D., D. D. S. By. It. G..
Ill Memoriam
-Henry Hobart Keecli,
M. D., D. D. S. By F. J. S. G.

				

